# Deep Residual Network Predicts Cortical Representation and Organization of Visual Features for Rapid Categorization

**DOI:** 10.1038/s41598-018-22160-9

**Published:** 2018-02-28

**Authors:** Haiguang Wen, Junxing Shi, Wei Chen, Zhongming Liu

**Affiliations:** 1 Weldon School of Biomedical Engineering, Purdue University, West Lafayette, IN USA; 2 School of Electrical and Computer Engineering, Purdue University, West Lafayette, IN USA; 3 Purdue Institute for Integrative Neuroscience, Purdue University, West Lafayette, IN USA; 4 Center for Magnetic Resonance Research, Department of Radiology, University of Minnesota Medical School, Minneapolis, MN USA

## Abstract

The brain represents visual objects with topographic cortical patterns. To address how distributed visual representations enable object categorization, we established predictive encoding models based on a deep residual network, and trained them to predict cortical responses to natural movies. Using this predictive model, we mapped human cortical representations to 64,000 visual objects from 80 categories with high throughput and accuracy. Such representations covered both the ventral and dorsal pathways, reflected multiple levels of object features, and preserved semantic relationships between categories. In the entire visual cortex, object representations were organized into three clusters of categories: biological objects, non-biological objects, and background scenes. In a finer scale specific to each cluster, object representations revealed sub-clusters for further categorization. Such hierarchical clustering of category representations was mostly contributed by cortical representations of object features from middle to high levels. In summary, this study demonstrates a useful computational strategy to characterize the cortical organization and representations of visual features for rapid categorization.

## Introduction

The visual cortex is capable of rapid categorization of visual objects^1,2^. This ability is attributable to cortical representation and organization of object information^3,4^. In the ventral temporal cortex, object representations are topologically organized^5^, spanning a high-dimensional space^6^ and being largely invariant against low-level appearance^1,7^. Knowledge about object categories is also represented in dorsal visual areas^8–10^ or even beyond the visual cortex^11^ where non-visual attributes of objects are coded^12,13^. In addition to their distributed representations^14,15^, object attributes are hierarchically organized and progressively emerge from visual input^4^. It is thus hypothesized that the brain categorizes visual objects based on their attributes represented in multiple stages of visual processing^5,13^.

To understand the basis of object categorization, it is desirable to map cortical representations of as many objects from as many categories as possible. The resulting maps provide the stimulus-response samples to address the representational structure that enables the brain to categorize or differentiate visual objects. Many studies have used functional magnetic resonance imaging (fMRI) to map brain activations with category-specific images^12,15–19^. Although such approaches are valuable for studying object categorization, it is expensive to cover many objects or categories in experiments, and it is arguably difficult to extrapolate experimental findings to new objects or categories. Moreover, object representations in the voxel space do not directly reveal the neural computation that give rise to such representations. It is also desirable to develop a model of hierarchical visual processing^20^ to be able to explain (or predict) cortical representations of visual objects with (or without) experimental data.

Advances in deep learning^21^ have established a range of deep neural networks (DNN) inspired by the brain itself^4,22^. Such models have been shown to be able to achieve human-level performance in object classification, segmentation, and tracking^21^. On the basis of DNNs, encoding models could be built to predict cortical responses to natural images^23–27^ or videos^28,29^. As the accuracies of predicted responses were high and robust in the entire visual cortex^29^, DNN-based encoding models are arguably advantageous than other models that only account for either the lowest^30,31^ or highest^32^ level of visual processing.

Recent studies also suggest that DNN-based encoding models may be generalized to new images or videos^24,25,27,29^. In this sense, the models provide a platform to simulate cortical representations of in principle infinite exemplars of a large number of object categories^27,29^, beyond what is experimentally attainable^17,33–36^. In addition, DNN views an image as a set of hierarchically organized features, rather than as a pixel array. The features are learned from millions of images to model image statistics in different levels of abstraction^21^. The learned features are much richer and more fine-grained than what may be intuitively defined (by humans) as the mid-level features. Through DNN-based encoding models, it is plausible to map object representations of specific features from each layer in DNN, allowing object categorization to be addressed at each level of visual processing.

Extending from recent studies^23–27,29^, we used a deep residual network (ResNet)^37^ to define, train, and test a generalizable, predictive, and hierarchical model of natural vision by using extensive fMRI data from humans watching >10 hours of natural videos. Taking this predictive model as a “virtual” fMRI scanner, we synthesized the cortical response patterns with 64,000 natural pictures including objects from 80 categories, and mapped cortical representations of these categories with high-throughput. We evaluated the category selectivity at every voxel in the visual cortex, compared cortical representational similarity with their semantic relationships, and evaluated the contributions from different levels of visual features to the cortical organization of categories. Consistent but complementary to prior experimental studies^12,15,16,32,38–43^, this study used a model-based computational strategy to study how cortical representations of various levels of object knowledge sub-serve categorization.

## Results

### ResNet predicted widespread cortical responses to natural visual stimuli

In line with recent studies^23–27,29^, we used a deep convolutional neural network to establish predictive models of cortical fMRI representations of natural visual stimuli. Specifically, we used ResNet – a deep residual network for computer vision^37^. With a much deeper architecture, ResNet offers more fine-grained layers of visual features, and it performs better in image recognition than similar but shallower networks, e.g. AlexNet^44^ as explored in prior studies^23–27,29,45^. In this study, we used ResNet to extract visual features from video stimuli, and used the extracted features to jointly predict the evoked fMRI response through a voxel-wise linear regression model. This encoding model was trained with a large amount of fMRI data during a training movie (12.8 hours for Subject 1, and 2.4 hours for Subject 2, 3), and tested with an independent testing movie (40 minutes).

**Figure 1:**
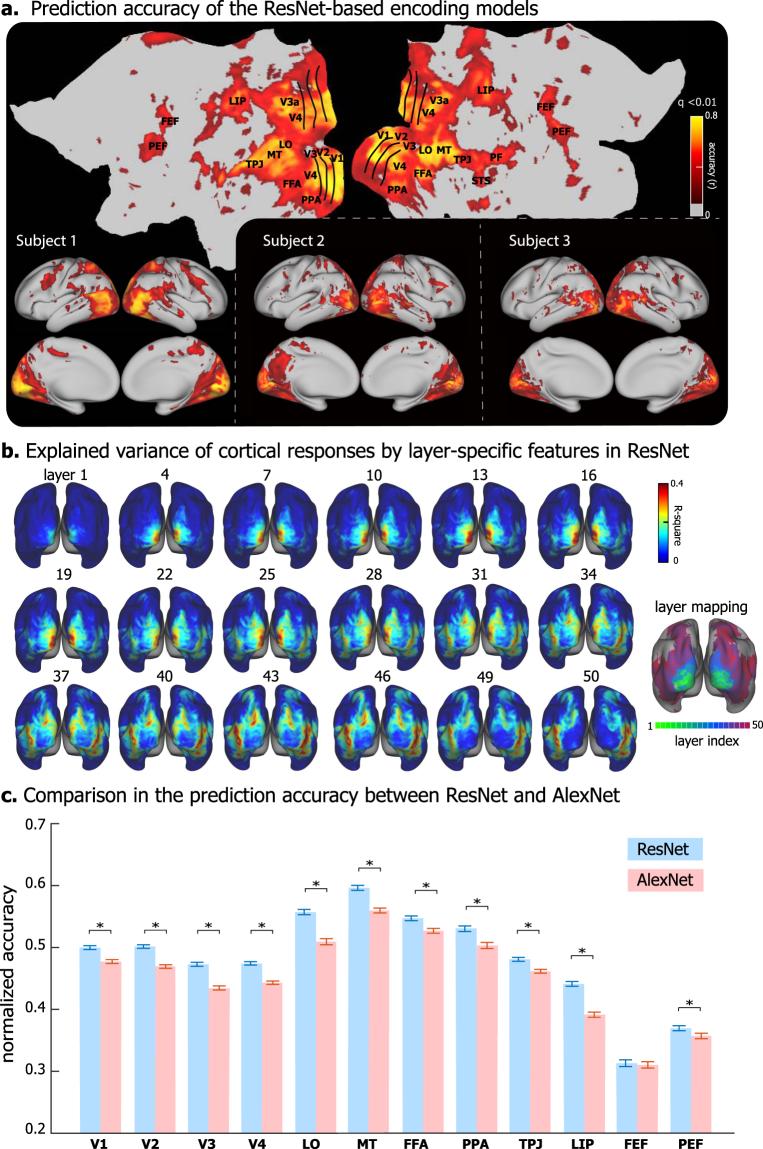
DNN-based Voxel-wise encoding models. **(a)** Performance of ResNet-based encoding models in predicting the cortical responses to novel testing movies for three subjects. The accuracy is measured by the average Pearson’s correlation coefficient (r) between the predicted and observed fMRI responses across five testing movies (q < 0.01 after correction for multiple comparison using the false discovery rate (FDR) method, and with threshold r > 0.2). The prediction accuracy is displayed on both flat (top) and inflated (bottom left) cortical surfaces for Subject 1. **(b)** Explained variance of the cortical response to testing movie by the layer-specific visual features in ResNet. The right shows the index to the ResNet layer that best explains the cortical response at every voxel. **(c)** Comparison between the ResNet-based and the AlexNet-based encoding models. Each bar represents the mean* ± *SE of the prediction accuracy (normalized by the noise ceiling, i.e. dividing prediction accuracy (r) by the noise ceiling at every voxel) within a ROI across voxels and subjects, and * indicates significance (p < 0.001) with paired t-test.

The encoding accuracy (i.e. the correlation between the predicted and measured fMRI signals during the testing movie) was overall high (r = 0.43* ± *0.14, 0.36* ± *0.12, and 0.37* ± *0.11 for Subject 1, 2 and 3, respectively) and statistically significant (permutation test, corrected at FDR q < 0.01) throughout the visual cortex in every subject (Fig. 1a). The encoding accuracy was comparable among the higher-order ventral-stream areas, e.g. fusiform face area (FFA) and parahippocampal place area (PPA), as well as early visual areas, e.g. V1, V2, and V3 (Fig. 1c). The accuracy was relatively lower at dorsal-stream areas such as lateral intraparietal area (LIP), frontal eye fields (FEF), parietal eye fields (PEF), but not the middle temporal area (MT) (Fig. 1c). Different cortical regions were preferentially correlated with distinct layers in ResNet. The lower to higher level visual features encoded in ResNet were gradually mapped onto areas from the striate to extrastriate cortex along both ventral and dorsal streams (Fig. 1b), in agreement with previous studies^25–29,45,46^. The prediction accuracy was consistently higher with (the deeper) ResNet than with (the shallower) AlexNet (Fig. 1c). These results suggest that the ResNet-based voxel-wise encoding models offer generalizable computational accounts for the complex and nonlinear relationships between natural visual stimuli and cortical responses at widespread areas involved in various levels of visual processing.

### Encoding models predicted cortical representations of various object categories

As explored before^27,29^, the voxel-wise encoding models constituted a high-throughput platform to synthesize cortical activations with an infinitely large number of natural pictures that are unrealistic or expensive to acquire with most experimental approaches. Here, we used this strategy to predict the pattern of cortical activation with each of the 64,000 natural pictures from 80 categories with on average 800 exemplars per category. By averaging the predicted activation maps across all exemplars of each category, the common cortical activation within this category was obtained to report its cortical representation.

**Figure 2:**
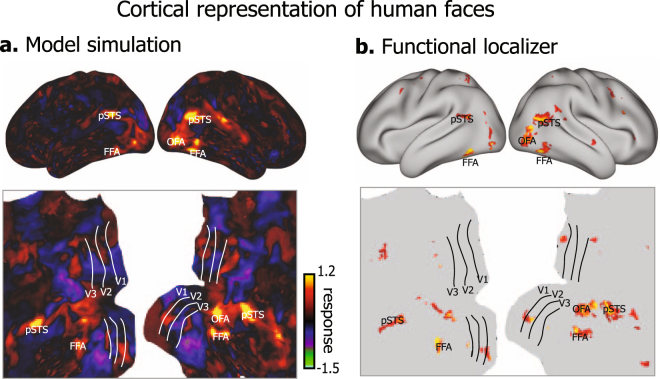
Human-face representations with encoding models and functional localizer. **(a)** Model-simulated representation of human face from ResNet-based encoding models. The representation is displayed on both inflated (top) and flat (bottom) cortical surfaces. **(b)** Face vs. non-face contrast map obtained with a face localizer experiment shows regions selective for human faces, including occipital face area (OFA), fusiform face area (FFA), and posterior superior temporal sulcus (pSTS).

**Figure 3:**
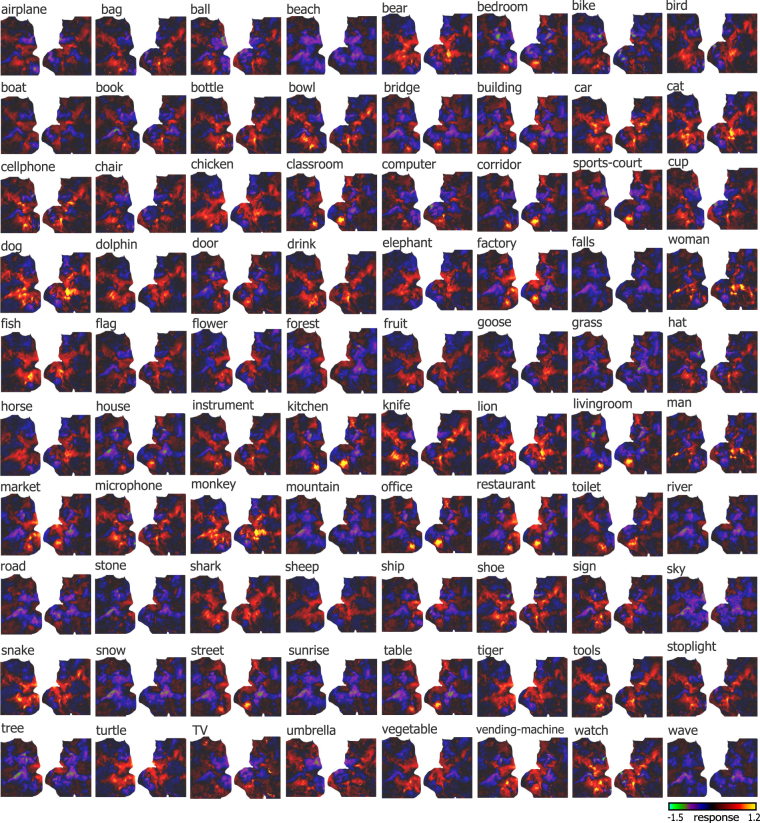
Cortical representations of 80 object categories. Each panel shows the representation map of an object category on flat cortical surface from Subject 1. The category label is on top left. The color bar shows the cortical response. Each map covers the same extent on the cortex as shown in Fig. 2a, bottom.

For example, averaging the predicted responses to various human faces revealed the cortical representation of the category “face” regardless of the position, size, color, angle, perspective of various faces (Fig. 2a). Such a model-simulated “face” representation was consistent with the fMRI-mapping result obtained with a block-design functional localizer that contrasted face vs. non-face pictures (Fig. 2b). In a similar manner, cortical representations of all 80 categories were individually mapped (Fig. 3). The resulting category representations were found not only along the ventral stream, but also along the dorsal stream albeit with relatively lower amplitudes and a smaller extent.

**Figure 4:**
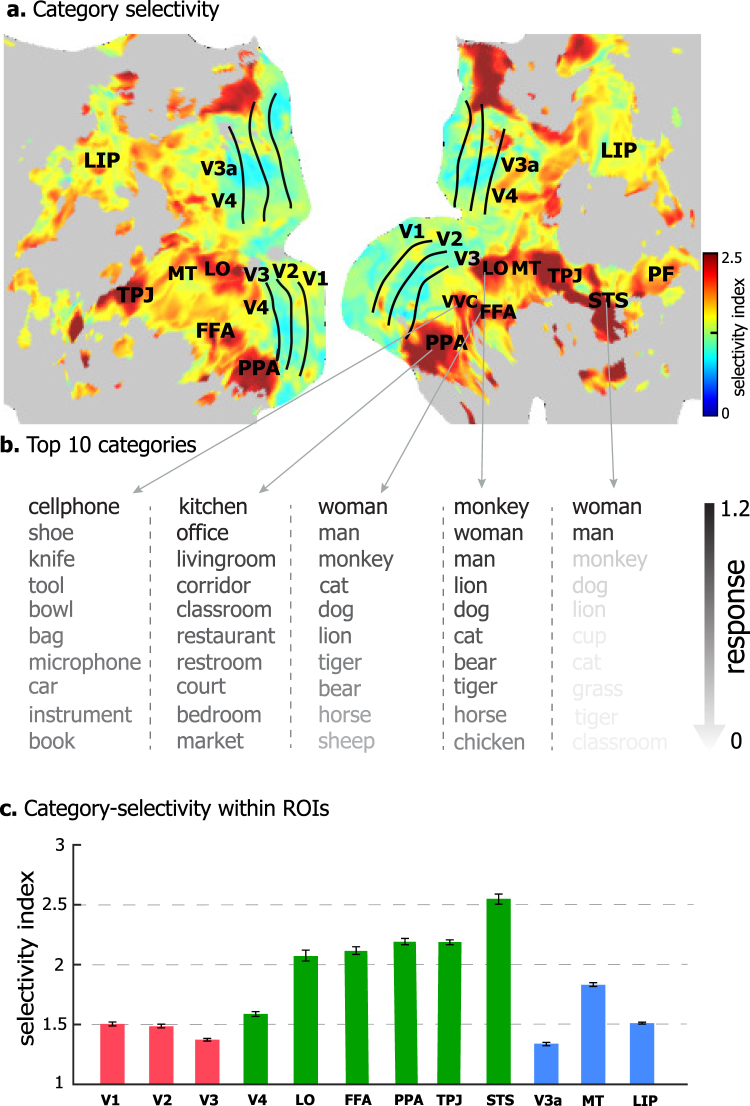
Category-selectivity at individual cortical locations. **(a)** The category-selectivity across the cortical surface. **(b)** The category-selectivity profile of example cortical locations. For each location, top 10 categories with the highest responses are showed in a descending order. **(c)** Category-selectivity within ROIs (mean ± SE) in the early visual areas (red), ventral stream areas (green), and dorsal stream areas (blue).

For each voxel, the model-predicted response as a function of category was regarded as the voxel-wise profile of categorical representation. The category selectivity – a measure of how a voxel was selectively responsive to one category relative to others^47^, varied considerably across cortical locations (Fig. 4a). Voxels with higher category selectivity were clustered into discrete regions including the bilateral PPA, FFA, lateral occipital (LO) area, the temporo-parietal junction (TPJ), as well as the right superior temporal sulcus (STS) (Fig. 4a). The profile of categorical representation listed in a descending order (Fig. 4b), showed that FFA, OFA, and pSTS were selective to humans or animals (e.g. man, woman, monkey, cat, lion); PPA was highly selective to places (e.g. kitchen, office, living room, corridor); and the ventral visual complex (VVC) was selective to man-made objects (e.g. cellphone, tool, bowl, car). In general, the ventral stream tended to be more category-selective than early visual areas (e.g. V1, V2, V3) and dorsal-stream areas (e.g. MT, LIP) (Fig. 4c).

### Distributed, overlapping, and clustered representations of categories

**Figure 5:**
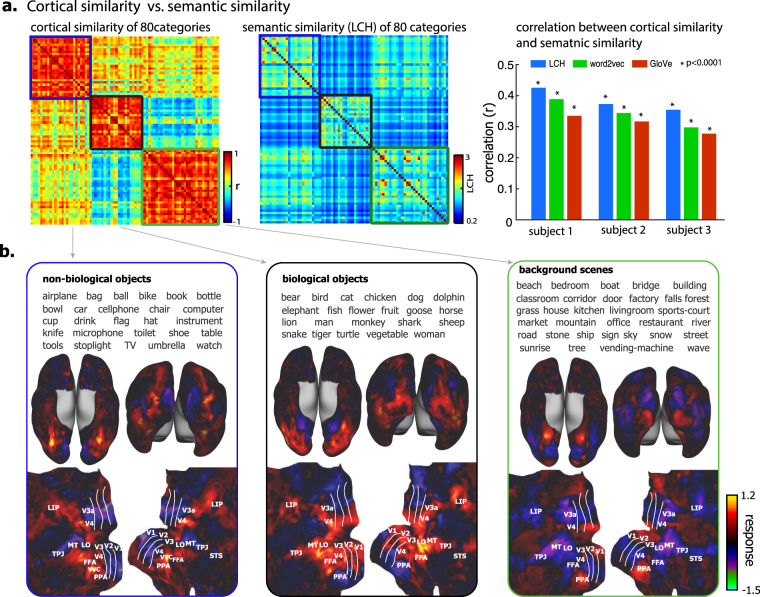
Categorical similarity and clustering in cortical representation at the scale of the entire visual cortex. **(a)** The left is the (inter-category) similarity matrix (Pearson’s correlation r) of cortical representation. Each element represents the cortical similarity between a pair of categories averaged across subjects (see individual results in Supplementary Fig. S2). It is well separated into three clusters with modularity Q = 0.35. The middle is the (inter-category) similarity matrix of semantic meaning (measured by LCH). The right is the Pearson’s correlation between the inter-category cortical similarity and the inter-category semantic similarity (with three different measures, i.e. the LCH similarity, the word2vec similarity, and the GloVe similarity). **(b)** The three clusters of cortical representation are related to three superordinate-level categories: non-biological objects, biological objects, and background scenes. The average cortical representations across categories within each cluster are shown on both inflated and flattened cortical surfaces.

Although some ventral-stream areas (e.g. PPA and FFA) were highly (but not exclusively) selective to a certain category, no category was represented by any single region alone (Fig. 3). As suggested previously^15^, object categories were represented distinctly by distributed but partially overlapping networks (see examples in Supplementary Fig. S1). In the scale of nearly the entire visual cortex that was predictable by the encoding models (Fig. 1a), the spatial correlations in cortical representation between different categories were shown as a representational similarity matrix (Fig. 5a). This matrix revealed a clustered organization: categories were clustered into three groups such that cortical representations were more correlated among categories within the same group than across different groups (Fig. 5a, left), and the degree of clustering (quantified as the modularity index, Q) was high (Q = 0.35). Interestingly, categories clustered together on the basis of their cortical representations tended to have higher conceptual similarities, or closer relationships between the corresponding category labels as measured by their Leacock-Chodorow (LCH) similarity in WordNet^48^ (Fig. 5a, middle), or by the cosine distance between their vector representations after word2vec^49^ or GloVe^50^ transformation (Supplementary Fig. S5). Regardless of the distinct methods for measuring the semantic similarity, there was a significant correlation between the similarity in cortical representation and the similarity in semantics across all pairs of categories (Fig. 5a, right). Moreover, we examined the category representations in a finer scale confined to individual visual areas (V1, V2, V3, LO, FFA, PPA). For each of these areas, we evaluated the correlation between representational similarity and semantic similarity across all pairs of categories. The correlation tended to increase from lower (e.g. V1) to higher (e.g. FFA/PPA) areas in the ventral stream (Supplementary Fig. S3). However, the correlation was significant (p < 0.0001, permutation test) not only in higher ventral-stream areas, but also in mid-level areas (e.g. LO) or even lower areas (V2, V3). In sum, categories with closer cortical representations tend to bear similar semantic meanings, in the spatial scale of the whole visual cortex as well as visual areas at different stages of visual processing.

The representational clusters in the entire visual cortex grouped basic-level categories into super-ordinate-level categories. The first cluster included non-biological objects, e.g. airplane, bottle and chair; the second cluster included biological objects, e.g. humans, animals, and plants; the third cluster included places and scenes (e.g. beach, bedroom) (Fig. 5b). The cortical representation averaged within each cluster revealed the general cortical representations of superordinate categories. As shown in Fig. 5b, non-biological objects were represented by activations in bilateral sub-regions of the ventral temporo-occipital cortex (e.g. VVC); biological objects were represented by activations in the lateral occipital cortex and part of the inferior temporal cortex (e.g. FFA) but deactivations in parahippocampal cortex (e.g. PPA); background scenes were represented by activations in PPA but deactivations in the lateral occipital complex, partly anti-correlated with the activations with biological objects. The spatial correlations between the cortical representations of biological objects and background scenes were on average –0.17 ± 0.29, which should be cautiously taken as a tendency of anti-correlation instead of strong evidence for precisely opposite patterns of representations of these two kinds of categories.

### Mid-level visual features primarily accounted for superordinate categorization

**Figure 6:**
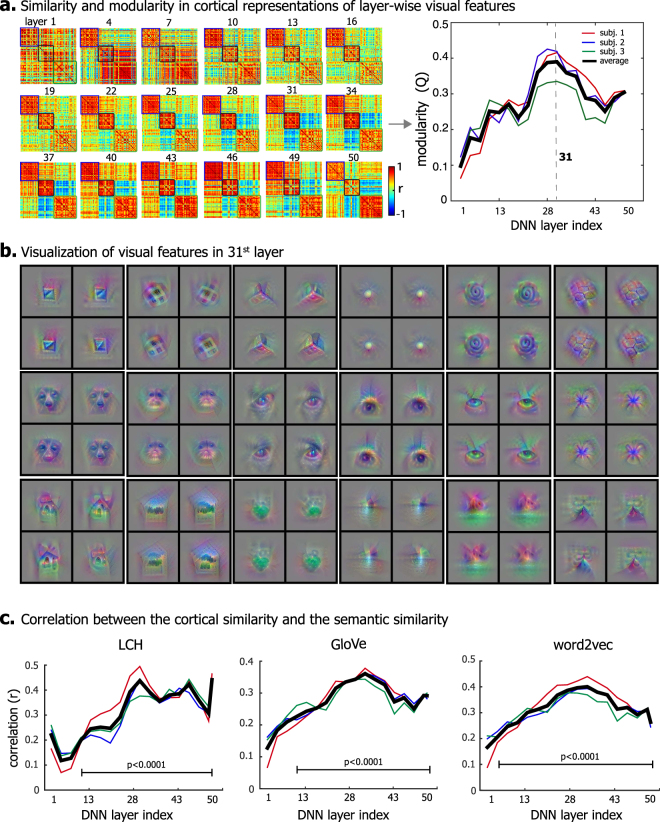
Contributions of different levels of visual features to the similarity and modularity in cortical representation. **(a)** The left shows the inter-category similarity of cortical representations contributed by layer-wise category information ranging from the lowest (layer 1) to highest (layer 50) layer. The order of categories is the same as in Fig. 6a. The right plot shows the corresponding modularity index due to visual features in each layer of ResNet. The visual features at the middle layers give rise to the highest modularity. **(b)** 18 example visual features at the 31^st^ layer are visualized in pixel space. Each visual feature shows 4 exemplars that maximize the feature representation. **(c)** The correlation between the inter-category cortical similarity across layers and the inter-category semantic similarity (with three different measures, i.e. the LCH similarity, the word2vec similarity, and the GloVe similarity) is shown for each layer in ResNet.

Which levels of visual features accounted for such a clustered organization of cortical category representation? To address this question, we simulated the cortical representation of single-layer features of every image exemplar in each category, by setting to zero all other layers in ResNet except one before inputting the feature representations into voxel-wise linear encoding models. Then we evaluated the similarity in cortical representation between categories at an increasing level of visual processing, progressively defined by the first through last layer in ResNet. Figure 6a (left) shows the representational similarity matrix attributed to features in each layer, thus decomposing the clustered organization in Fig. 5a by layers. In the earliest level of visual processing as specified by V1-like neurons in the first layer of ResNet, the similarity (or dissimilarity) among different categories was not apparent within (or across) the three superordinate categories (non-biological objects, biological objects, and background scenes). At layer 4, non-biological objects differed themselves from biological objects or background scenes, as the representational similarity appeared to reveal two clusters, rather than three clusters. Starting from layer 10 through 19, the three clusters emerged in the corresponding representational similarity matrices. Starting from layer 25, anti-correlations became clearly notable between the cluster of biological objects and the cluster of background scenes.

In a more quantitative way, we evaluated the modularity index of the three-cluster organization due to layer-wise features. Figure 6a (right) shows the modularity index as a function of the layer in ResNet. It suggests that the clustering of basic-level categories into superordinate categories emerged progressively and occurred in many levels of visual processing, while the clustering was the most apparent in the middle level (i.e. layer 31 in ResNet). To gain intuition about the types of visual information from the 31^st^ layer, the features encoded by individual units in this layer were visualized. Figure 6b illustrates the visualizations of some example features, showing shapes or patterns (both 2-D and 3-D), animal or facial parts (e.g. head and eye), scenic parts (e.g. house and mountain). Beyond these examples, other features were of similar types.

In addition, we evaluated the correlation between the inter-category semantic similarity and the corresponding similarity in cortical representation of the features in each layer. It turned out that the layer-wise correlations were significant (p < 0.001) for middle and high-level features, and the greatest correlation was not necessarily in the highest layer, but in the middle layer (around layer 31) (Fig. 6c). It suggests that semantic relationships emerge from object attributes in different levels of visual processing, and that the mid-level attributes (e.g. object shapes or parts) contribute the most to superordinate-level categorization.

### Clustered organization of cortical representation within superordinate categories

**Figure 7:**
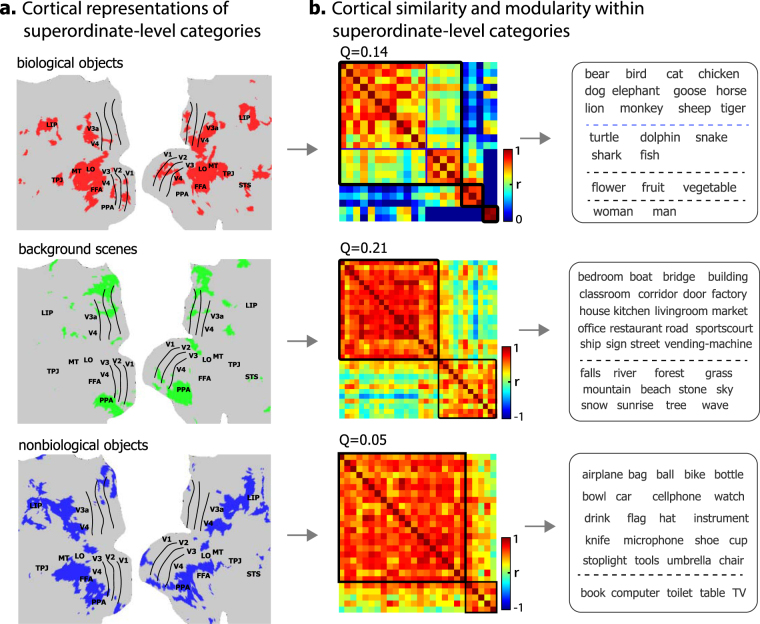
Categorical similarity and clustering in cortical representation within superordinate-level categories. **(a)** Fine-scale cortical areas specific to each superordinate-level category: biological objects (red), background scenes (green) and non-biological objects (blue). **(b)** The cortical similarity between categories in fine-scale cortical representation. The categories in each sub-cluster were displayed on the right. See individual results in Supplementary Fig. S2.

We further asked whether the similarly clustered organization could be extended to a lower level of categorization. That is, whether object representations were organized into sub-clusters within each superordinate-level cluster. For this purpose, we confined the scope of analysis from the whole visual cortex to finer spatial scales highlighted by the co-activation patterns within biological objects, non-biological objects, or background scenes (Fig. 7a). For example, within the regions where biological objects were represented (Fig. 7a, top), the representational patterns were further clustered into four sub-clusters: terrestrial animals, aquatic animals, plants, and humans (Fig. 7b, top). Similarly, the fine-scale representational patterns of background scenes were clustered into two sub-clusters corresponding to artificial (e.g. bedroom, bridge, restaurant) and natural scenes (e.g. falls, forest, beach) (Fig. 7, middle). However, the two clusters of non-biological objects did not bear any reasonable conceptual distinction (Fig. 7, bottom).

**Figure 8:**
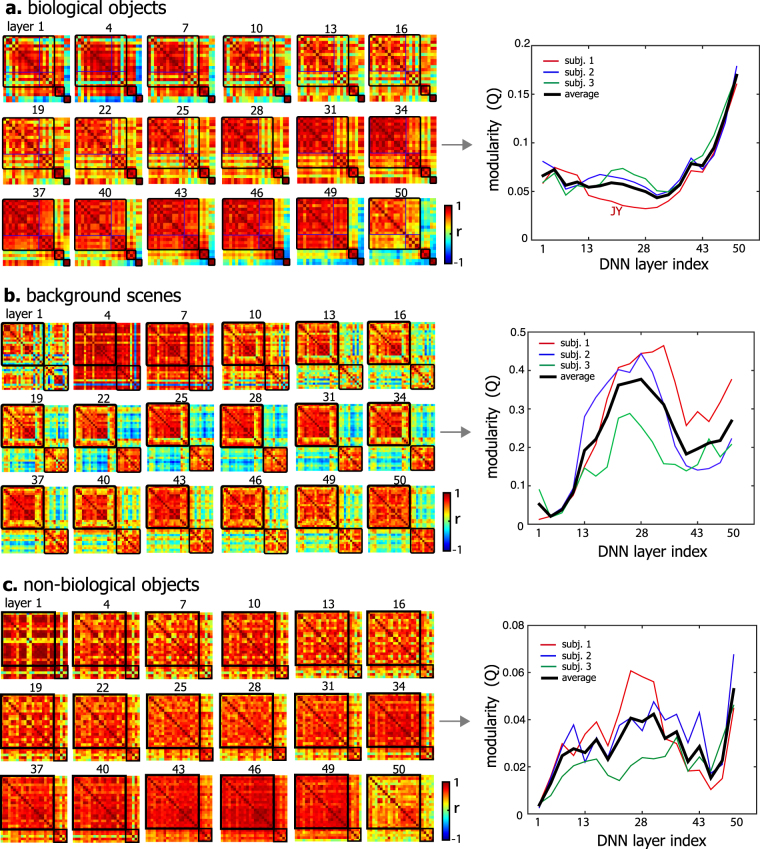
Contribution of layer-wise visual features to the similarity and modularity in cortical representations within superordinate-level categories. The left shows the similarity between categories in fine-scale cortical representations that are contributed by separated category information from individual layers. The order of categories is the same as in Fig. 7. The right plot shows the modularity index across all layers. The highest-layer visual features show the highest modularity for biological objects.

We also evaluated the contribution of layer-wise visual features to the fine-scale representational similarity and clustering. For biological objects, the modularity index generally increased from the lower to higher layer, reaching the maximum at the highest layer (Fig. 8a). Note that the highest layer encoded the most abstract and semantically relevant features, whose visualizations revealed the entire objects or scenes (see Supplementary Fig. S4) rather than object or scenic parts (Fig. 6b). In contrast, the modularity index reached the maximum at the 28^th^ layer for background scenes (Fig. 8b), but was relatively weak and less layer-dependent for non-biological objects (Fig. 8c).

## Discussion

This study demonstrates a high-throughput computational strategy to characterize hierarchical, distributed, and overlapping cortical representations of visual objects and categories. Results suggest that information about visual-object category entails multiple levels and domains of features represented by distributed cortical patterns in both ventral and dorsal pathways. Categories with similar cortical representations are more semantically related to one another. In a large scale of the entire visual cortex, cortical representations of objects are clustered into three superordinate categories (biological objects, non-biological objects, and background scenes). In a finer spatial scale that is specific to each cluster, cortical representations are organized into sub-clusters for finer categorization, e.g. biological objects are categorized into terrestrial animals, aquatic animals, plants, and humans. The clustered organization of cortical representation is more observable for object features in middle and high levels of complexity compared to low-level features. Therefore, the brain categorizes visual objects through the hierarchically clustered organization of object attributes emerging from various levels of visual processing, rather than any operation that only occurs at the highest level of the ventral-stream hierarchy.

Central to this study is the use of the categorization-driven deep ResNet for synthesizing the cortical representations of thousands of natural visual objects from many categories. This strategy has a much higher throughput in sampling a virtually infinite number of exemplars of visual objects^27,29^, compared to prior studies that are limited to fewer categories with much fewer exemplars per category^17,33–36^. The sample size could be further extendable, since the ResNet-based encoding models presumably account for the relationships between cortical responses and visual features that are generalizable to different and new natural images, objects, and categories beyond which the models have been explicitly trained with. The model predictions are highly accurate and consistent with experimentally observed cortical responses to video stimuli and cortical representations to specific objects (e.g. human faces). The encoding accuracy may be further improved given an even larger and more diverse video-fMRI dataset to train the model, and a more biologically relevant deep neural net that better matches the brain and better performs in computer-vision tasks^24^. In this sense, the encoding models in this study are based on so far the largest video-fMRI training data from single subjects; and ResNet also outperforms AlexNet in categorizing images^37,44^ and predicting the brain (Fig. 1c). The encoding models reported here are thus arguably more powerful for predicting and mapping hierarchical cortical representations in the entire visual cortex, compared to other conceptually similar models in prior studies^23–27,29^.

What is also advantageous is that ResNet decomposes category information into multiple layers of features progressively emerging from low to middle to high levels. As such, ResNet offers a computational account of hierarchical cortical processing for categorization, yielding quantitative description of every object or category in terms of different layers of visual features. Mapping the layer-wise features from the ResNet onto the brain helps to address what drives the cortical organization of object knowledge and supports various levels of categorization.

The ResNet is trained with large-scale image set (~1.3 million natural images) for recognizing 1,000 visual object categories^37^. Though specific categories are used in training the ResNet, the trained model is generalizable enough to represent the semantics in our training and testing stimuli, and is transferrable for recognizing new categories based on the generic representations in the learned feature space for transfer learning^51,52^. The generalizability of the feature space enables prediction of the cortical representations of a wide range of categories far beyond what the network has been explicitly trained with. For example, the model is able to predict the face representation even though the ResNet is not trained for recognizing faces (Fig. 2).

Our results support the notion that visual-object categories are represented by distributed and overlapping cortical patterns^15^ rather than clustered regions^16,38,39^. Given this notion, the brain represents a category not as a single entity but a set of defining attributes that span multiple domains and levels of object knowledge. Different objects bear overlapping representational patterns that are both separable and associable, allowing them to be recognized as one category in a particular level, but as different categories in another level. For example, a lion and a shark are both animals but can be more specifically categorized as terrestrial and aquatic animals, respectively. The distributed and overlapping object representations, as weighted spatial patterns of attribute-based representations^12^, constitute an essential principle underlying the brain’s capacity for multi-level categorization.

Category representations may become highly selective at spatially clustered regions^16,38,39^. The category-selective regions are mostly in the ventral temporal cortex (Fig. 4), e.g. the FFA, PPA, and LO. The existence of category-selective regions does not contradict with distributed category representation. Instead, a region specific to a given category is thought to emerge from its connectivity with other locations that represent the defining attributes of that category^53^, or subserve the category-specific action and cognition^54^.

The cortical representational similarity between different categories is highly correlated with their semantic relationship (Fig. 5). In other words, the semantic relationship is preserved by cortical representation. This finding lends support for the notion of a continuous semantic space underlying the brain’s category representation^32^, which is a parsimonious hypothesis to bridge neural representation and linguistic taxonomy^55^. However, category information is not limited to semantic features, but includes hierarchically organized attributes, all of which define categories and their conceptual relationships. For example, “face” is not an isolated concept; it entails facial features (“eyes”, “nose”, “mouth”), each also having its own defining features. The similarity and distinction between categories may be attributable to one or multiple levels of features. In prior studies^32^, the hierarchical nature of category information is not considered as every exemplar of each category is annotated by a pre-defined label. This causes an incomplete account of category representation, leaving it difficult to disentangle the various levels of category information that may be used to associate or distinguish categories.

We have overcome this limit by disentangling multiple layers of features from visual objects and evaluating their respective cortical representations. Our results show that different levels of features make distinctive contributions to the clustering of category representation in the visual cortex. Coarse categories (i.e. biological objects, non-biological objects, and background scenes) are most attributable to mid-level features, e.g. shapes, textures, and object parts (Fig. 6). In a finer level of categorization, terrestrial animals, aquatic animals, plants, and humans are most distinguishable in the semantic space; categorization of man-made and natural scenes is most supported by mid-level features (Fig. 8). In addition, the semantic similarity between categories is correlated with the spatial similarity in cortical representation of their middle to high-level visual features (Fig. 6), not necessarily confined to one level or domain of features or a single cortical region, e.g. ITC^41^. Recent studies have also shown that the cortical organization of visual objects may be explained in part by similarity in low-level visual features^56–58^, shape^57,59–64^, and the real-word or conceptual size of objects^65,66^. This study further expands the dimension of visual or conceptual features beyond what can be intuitively defined^67^, by using data-driven features extracted from ResNet^37^,

This study is focused on the use of CNN-based encoding models to study the brain’s mechanism for categorization, rather than only on the validation of a CNN against neuroscience data. Arguably, if a model is able to predict cortical responses to natural visual stimuli, it is reasonable to use the model as a computational tool to characterize the brain itself. Similar ideas have been utilized to map the brain’s semantic representation by using semantics-based encoding models^32^, yielding insightful findings about how the brain represents natural language. However, it should be noted that although it is successful explaining significant variance of cortical responses to video stimuli, ResNet is not a perfect model of the visual cortex, and does not reach the noise ceiling. ResNet, or other types of feed-forward-only CNN, ignores the temporal relationships between video frames. Thus, the ResNet-based encoding models are more suitable to be trained with well-separated static image stimuli, which would take much longer time to acquire an equivalent amount of training data (as with video stimuli) for training the encoding models with millions of parameters. In addition, ResNet does not include any feedback connections or account for active attention, and fails to mimic the brain’s ability of unsupervised learning^68^. For these reasons, ResNet is by no means an ultimate model of the visual cortex. Nevertheless, a feed-forward CNN is appropriate for modeling the brain’s mechanism for rapid visual categorization, which is arguably mostly feed-forward^1,69^. Our results suggest that CNN can be used to reproduce the cortical organization of category representations, selectivity, and clustering, which often require extensive experimental efforts to reveal^17,33–36^. The CNN-based encoding models may allow researchers (or students) to run “virtual-fMRI” experiments with arbitrary visual stimuli, simulate cortical activations, and accordingly raise hypotheses for testing with real experiments. In the meantime, it awaits future studies to validate this strategy with more experimental data and a rich stimulus set with different configurations, and to develop more biologically plausible models to replace CNN in this computational strategy.

## Materials and Methods

### Experimental data

We used and extended the human experimental data from our previous study^29^, according to experimental protocols approved by the Institutional Review Board at Purdue University with informed consent from all human subjects prior to their participation. All methods were performed in accordance with the relevant guidelines and regulations. Briefly, the data included the fMRI scans from three healthy subjects (Subject 1, 2, 3, all female) when watching natural videos. For each subject, the video-fMRI data were split into two independent datasets: one for training the encoding model and the other for testing it. For Subject 2 & 3, the training movie included 2.4 hours of videos; the testing movie included 40 minutes of videos; the training movie was repeated twice, and the testing movie was repeated ten times. For Subject 1, the training movie included not only those videos presented to Subject 2 and 3, but also 10.4 hours of new videos. The new training movie was presented only once. The movie stimuli included a total of ~9,300 video clips manually selected from *YouTube*, covering a variety of real-life visual experiences. All video clips were concatenated in a random sequence and separated into 8-min sessions. Every subject watched each session of videos (field of view: 20.3° × 20.3°) through a binocular goggle with the eyes fixating at a central cross (0.8° × 0.8°). During each session, whole-brain fMRI scans were acquired with 3.5 mm isotropic resolution and 2 s repetition time in a 3-T MRI system by using a single-shot, gradient-recalled echo-planar imaging sequence (38 interleaved axial slices with 3.5 mm thickness and 3.5 × 3.5 mm^2^ in-plane resolution, TR = 2000 ms, TE = 35 ms, flip angle = 78°, field of view = 22 × 22 cm^2^). Structural MRI data with T_1_ and T_2_ weighted contrast were also acquired with 1 mm isotropic resolution for every subject. The volumetric fMRI data were preprocessed and co-registered onto a standard cortical surface template^70^. For each cortical location, the 4^th^-order polynomial trend was removed from the fMRI signal. For training and testing encoding models (as described latter), the fMRI signals were averaged over repetitions if there were multiple repeats and then standardized (i.e. remove the mean and normalize the variance). More details about the movie stimuli, data preprocessing and acquisition are described elsewhere^29^.

### Deep residual network

In line with previous studies^23–27,29,45^, a feedforward deep neural network (DNN) was used to model the cortical representations of natural visual stimuli. Here, we used a specific version of the DNN known as the deep residual network (ResNet), which had been pre-trained to categorize natural pictures with the state-of-the-art performance^37^. In the ResNet, 50 hidden layers of neuron-like computational units were stacked into a bottom-up hierarchy. The first layer encoded location and orientation-selective visual features, whereas the last layer encoded semantic features that supported categorization. The layers in between encoded increasingly complex features through 16 residual blocks; each block included three successive layers and a shortcut directly connecting the input of the block to the output of the block^37^. Compared to the DNNs in prior studies^24–26,29,45,71^, the ResNet was much deeper and defined more fine-grained hierarchical visual features. The ResNet could be used to extract feature representations from any input image or video frame by frame. Passing an image into the ResNet yielded an activation value at each unit. Passing a video yielded an activation time series at each unit as the fluctuating representation of a given visual feature in the video.

### Encoding models

For each subject, we trained an encoding model to predict each voxel’s fMRI response to any natural visual stimuli^72^, using a similar strategy as previously explored^25,27,29^. The voxel-wise encoding model included two parts: the first part was nonlinear, converting the visual input from pixel arrays into representations of hierarchical features through the ResNet; the second part was linear, projecting them onto each voxel’s fMRI response. The encoding model used the features from 18 hidden layers in the ResNet, including the first layer, the last layer, and the output layer for each of the 16 residual blocks. For video stimuli, the time series extracted by each unit was convolved with a canonical hemodynamic response function (HRF) with the peak response at 4 s, and down-sampled to match the sampling rate of fMRI, and then standardized (i.e. remove the mean and normalize the variance).

The feature dimension was reduced by applying principle component analysis (PCA) first to each layer and then to all layers in ResNet. The principal components of each layer were a set of orthogonal vectors that explained > 99% variance of the layer’s feature representations given the training movie. The layer-wise dimension reduction was expressed as equation (1).

1 $${{\boldsymbol{f}}}_{l}({\bf{x}})=\,{{\boldsymbol{f}}}_{l}^{o}({\bf{x}}){{\bf{B}}}_{l}$$

where ${{\boldsymbol{f}}}_{l}^{o}({\bf{x}})$ (1 × *p*_*l*_) is the original feature representation from layer *l* given a visual input **x**, **B**_*l*_ (*p*_*l*_ × *q*_*l*_) consists of unitary columnar vectors that represented the principal components for layer *l*, ${{\boldsymbol{f}}}_{l}({\bf{x}})$ (1 × *q*_*l*_) is the feature representation after reducing the dimension from *p*_*l*_ to *q*_*l*_.

Following the layer-wise dimension reduction, the feature representations from all layers were further reduced by using PCA to retain > 99% variance across layers. The final dimension reduction was implemented as equation (2).

2 $${\boldsymbol{f}}({\bf{x}})={{\boldsymbol{f}}}_{1:L}({\bf{x}}){{\bf{B}}}_{1:L}$$

where ${{\boldsymbol{f}}}_{1:L}({\bf{x}})=[\frac{{{\boldsymbol{f}}}_{1}({\bf{x}})}{\sqrt{{p}_{1}}},\,\ldots \,,\frac{{{\boldsymbol{f}}}_{L}({\bf{x}})}{\sqrt{{p}_{L}}}]$ is the feature representation concatenated across *L* layers, ${{\bf{B}}}_{1:L}$ consists of unitary principal components of the layer-concatenated feature representations of the training movie, and ${\boldsymbol{f}}({\bf{x}})$ (1 × *k*) is the final dimension-reduced feature representation.

For the second part of the encoding model, a linear regression model was used to predict the fMRI response ${r}_{v}({\bf{x}})$ at voxel $v$ evoked by the stimulus ${\bf{x}}$ based on the dimension-reduced feature representation ${\boldsymbol{f}}({\bf{x}})$ of the stimulus, as expressed by equation (3).

3 $${r}_{v}({\bf{x}})={\boldsymbol{f}}({\bf{x}})\,{{\bf{w}}}_{v}+{\varepsilon }_{v}$$

where ${{\bf{w}}}_{v}$ is a columnar vector of regression coefficients specific to voxel $v$, and ${\varepsilon }_{v}$ is the error term. As shown in equation (4), L_2_-regularized least-squares estimation was used to estimate ${{\bf{w}}}_{v}$ given the data during the training movie (individual frames were indexed by *i* = , …, *N*), where the regularization parameter was determined based on nine-fold cross-validation.

4 $$\mathop{\,{\hat{{\bf{w}}}}_{v}={\rm{\arg }}\,{\rm{\min }}}\limits_{\,{{\bf{w}}}_{v}}\frac{1}{N}\sum _{i=1}^{N}{({r}_{v}({{\bf{x}}}_{i})-{\boldsymbol{f}}({{\bf{x}}}_{i}){{\bf{w}}}_{v})}^{2}+\lambda {\Vert {{\bf{w}}}_{v}\Vert }_{2}^{2}$$

After the above training, the voxel-wise encoding models were evaluated for their ability to predict the cortical responses to the novel testing movie (not used for training). The prediction accuracy was quantified as the temporal correlation (*r*) between the predicted and observed fMRI responses at each voxel given the testing movie. Since the testing movie included five distinct sessions, the prediction accuracy was evaluated separately for each session, and then averaged across sessions. The significance of the voxel-wise prediction accuracy was evaluated with a block-permutation test^73^ (corrected at false discovery rate (FDR) *q* < 0.01), as used in our prior study^29^.

We also evaluated the correspondence between the hierarchical layers in ResNet and the hierarchical cortical areas underlying different stages of visual processing, in line with previous studies^23–29,45^. For this purpose, we calculated the variance of the response at a voxel explained by the visual features in single layers. Specifically, the features extracted from the testing movie were kept only for one layer in the ResNet, while setting to zeros for all other layers. Through the voxel-wise encoding model, the variance (measured by R-squared) of the response explained by the single layer was calculated. For each voxel, we identified the best corresponding layer with the maximum explained variance and assigned its layer index to this voxel. The assigned layer index indicated the processing stage this voxel belonged to.

We also tested whether the deeper ResNet outperformed the shallower AlexNet^44^ in predicting cortical responses to natural movies, taking the latter as the benchmark given its state-of-the-art encoding performance in prior studies^25,26,29^. For this purpose, we trained and tested similar encoding models based on the AlexNet with the same analysis of the same dataset. We compared the prediction accuracy between ResNet and AlexNet for regions of interest (ROIs) defined in an existing cortical parcellation^74^, and further evaluated the statistical significance of their difference using a paired t-test (p < 0.001) across all voxels within each ROI. Considering the noise in the data, we also calculated the noise ceiling of the predictability at each voxel. The noise ceiling indicated the maximum accuracy that a model could be expected to achieve given the level of noise in the testing data^75^. The noise and signal in fMRI were assumed to follow Gaussian distribution and the mean of noise was zero. For each testing session, we estimated the noise level and the mean/SD of the signal for every voxel. We used Monte Carlo simulation to obtain the noise ceiling. For each simulation, we generated a signal from the signal distribution, and generated a noisy data by adding the signal and the noise drawn from the noise distribution, and calculated the correlation between the signal and the data. We performed 1,000 simulations for each testing session, and took the median correlation as the noise ceiling. The ceiling was then averaged across sessions.

### Human-face representations with encoding models and functional localizer

The ResNet-based encoding models were further used to simulate cortical representations of human faces, in comparison with the results obtained with a functional localizer applied to the same subjects. To simulate the cortical “face” representation, 2,000 human-face pictures were obtained by Google Image search. Each of these pictures was input to the voxel-wise encoding model, simulating a cortical response map as if it were generated when the subject was actually viewing the picture, as initially explored in previous studies^27,29^. The simulated response maps were averaged across all the face pictures, synthesizing the cortical representation of human face as an object category.

To validate the model-synthesized “face” representation, a functional localizer^76^ was used to experimentally map the cortical face areas on the same subjects. Each subject participated in three sessions of fMRI with a randomized block-design paradigm. The paradigm included alternating ON-OFF blocks with 12 s per block. During each ON block, 15 pictures (12 novel and 3 repeated) from one of the three categories (face, object, and place) were shown for 0.5 s per each picture with a 0.3 s interval. The ON blocks were randomized and counter-balanced across the three categories. Following the same preprocessing as for the video-fMRI data, the block-design fMRI data were analyzed with a general linear model (GLM) with three predictors, i.e. face, object, and place. Cortical “face” areas were localized by testing the significance of a contrast (face > object and face > place) with p < 0.05 and Bonferroni correction.

### Synthesizing cortical representations of different categories

Beyond the proof of concept with human faces, the similar strategy was also extended to simulate the cortical representations of 80 categories through the ResNet-based encoding models. The category labels were shown in Fig. 3. These categories were mostly covered by the video clips used for training the encoding models. For each category, around 800 pictures were obtained by Google Image search with the corresponding label, and were visually inspected to replace any exemplar that belonged to more than one category. There was a total of 64,000 objects from 80 categories. The cortical representation of each category was generated by averaging the model-simulated response map given every exemplar within the category.

We focused on cortical representations of basic-level object categories, as opposed to individual images. Although the models were able to simulate and characterize cortical activations with each of the images, as already done in our prior study^29^, herein the total number of images (64,000) was too large. This choice was also given our primary interest in representations of object knowledge, regardless of the luminance, position, and size of any object. However, the exclusive focus on category-average representations, may be biased by how categories were defined and how images were selected (by humans). More detailed analysis of responses to individual image exemplars is helpful to mitigate this bias or ambiguity^17^.

### Category selectivity

Following the above analysis, cortical representations were compared across categories to quantify the category selectivity of various locations and ROIs. For each voxel, its selectivity to category $i$ against other categories ${i}^{c}$ was quantified with equation (5), as previously suggested^47^.

5 $${d}_{i}^{\text{'}}=\frac{{\bar{r}}_{i}-{\bar{r}}_{{i}^{c}}}{\sqrt{({\sigma }_{i}^{2}+{\sigma }_{{i}^{c}}^{2})/2}}$$

where ${\bar{r}}_{i}$ and ${\sigma }_{i}^{2}$ are the mean and variance of the responses to the exemplars in category $i$, and ${\bar{r}}_{{i}^{c}}$ and ${\sigma }_{{i}^{c}}^{2}$ were counterparts to all exemplars in other categories ${i}^{c}$. Irrespective of any specific category, the general category-selectivity for each voxel was its maximal *d*′ index among all categories, i.e. $d^{\prime} =\mathop{{\rm{\max }}}\limits_{i}\{{d}_{i}^{\text{'}}\}$. A *d*′ index of zero suggests non-selectivity to any category, and a higher *d*′ index suggests higher category-selectivity. The category selectivity of any given voxel was also inspected by listing the categories in a descending order of their representations at the voxel. We also obtained the ROI-level category selectivity by averaging the voxel-wise selectivity across voxels and subjects. ROIs were defined in an existing cortical parcellation^74^.

### Categorical similarity and clustering in cortical representation

To reveal how the brain organizes categorical information, we assessed the similarity (i.e. the Pearson’s correlation of the spatial response patterns across the predictable voxels with q < 0.01 in permutation test and prediction accuracy r > 0.2) in cortical representations between categories. Based on such inter-category similarity, individual categories were grouped into clusters using k-means clustering^77^. The goodness of clustering was measured as the modularity index, which quantified the inter-category similarities within the clusters relative to those regardless of the clusters^78^. The number of clusters was determined by maximizing the modularity index. To quantify the modularity index, the categorical similarity was viewed as a signed, weighted, and undirected network^78^. Each node represented one category, and each weighted edge represented the similarity between two categories. The modularity was then measured as the probability of having edges falling within clusters in the network against a random network (null case) with the same number of nodes and edges placed at random preserving the degree of each node. Specifically, given a *positive* weighted matrix ***S*** (*S*_*ij*_ denotes the weight between categories *i* and *j*, and $S=2\sum _{i}\sum _{j}{S}_{ij}$ denotes the double total weight), the modularity index *Q* was defined as $Q=\sum _{i}\sum _{j}({p}_{ij}-{q}_{ij}){\rm{\delta }}({C}_{i},{C}_{j})$, where *p*_*ij*_ = *S*_*ij*_/*S* is the probability of connecting category *i* and *j* in the network with edge weight *S*_*ij*_, ${q}_{ij}=(\sum _{j}{S}_{ij}/S)(\sum _{i}{S}_{ij}/S)$ denotes the expected probability of having edge between *i* and *j* in random networks, and ${\rm{\delta }}({C}_{i},{C}_{j})$ is the Kronecker delta function with value 1 if *i* and *j* are in the same cluster and 0 otherwise. Since the correlation coefficients ranged from −1 to 1, we separated the positive and negative weights by ${S}_{ij}={S}_{ij}^{+}-{S}_{ij}^{-}$ where ${S}_{ij}^{+}=\,{\rm{\max }}\{0,{S}_{ij}\}$ and ${S}_{ij}^{-}=\,{\rm{\max }}\{0,-{S}_{ij}\}$, and calculated their corresponding modularity *Q*^+^ and *Q*^−^. Then the total modularity was quantified as $Q=\frac{{S}^{+}}{{S}^{+}+{S}^{-}}{Q}^{+}-\frac{{S}^{-}}{{S}^{+}+{S}^{-}}{Q}^{-}$. The significance of the modularity index was assessed by permutation test against the null distribution obtained from shuffling the pair-wise similarities randomly for 100,000 times. The larger modularity means the larger deviation from the null case and the better differentiation between clusters. Noted that higher similarity within clusters and less similarity across clusters gives larger modularity.

The similarity in cortical representation between different categories was compared with their similarity in semantic meaning. Here, we explored three different models to measure the semantic similarity between categories. For the first model, the semantic similarity between categories was evaluated as the Leacock-Chodorow similarity (LCH)^48^ between the corresponding labels based on their relationships defined in the WordNet^79^ – a directed graph of words (as the nodes) and their *is-a* relationships (as the edges). Briefly, LCH computes the similarity (*s*) between two labels based on the shortest path (*p*) that connects the labels in the taxonomy and the maximum depth (*d*) of the taxonomy in which the labels occur through $s=-\mathrm{log}(p/2d)$. The second model was the word2vec model that represented text words in a continuous vector space that captured a large number of precise syntactic and semantic word relationship^49^. We used the published model that was pretrained by Google on 100 billion words from Google News. The model was trained to accurately predict surrounding words given the current word. We used it to transform the category labels to vectors, and then calculated the semantic similarity between labels as the cosine distance of their corresponding vectors. The third model was the GloVe model that also represented words in vectors and captured fine-grained semantic and syntactic regularities using vector arithmetic^50^. GloVe was trained on global word-word co-occurrence statistics from a corpus of text. Similarly, we used the pretrained GloVe (trained on a large corpus including 840 billion tokens) to derive the vectors of category labels and calculate their semantic similarity as the cosine distance between the vectors. After obtaining the inter-category semantic similarity (LCH, word2vec, or GloVe), we evaluated the Pearson’s correlation between the cortical and semantic similarities. Before computing the correlation, the cortical similarity was transformed to z-score by using the Fisher’s z-transformation. Since the similarity was symmetric, the correlation was computed over the values in the upper (or equivalently the lower) triangular region of the similarity matrix^80^. The significance was assessed by random permutation of the category labels (i.e. reordering rows and columns of the cortical similarity matrix according to this permutation and computing the correlation). By repeating the permutation step 10,000 times, we obtained a distribution of correlations simulating the null hypothesis that the two similarity matrices are unrelated^80^.

### Layer-wise contribution to cortical categorical representation

We also asked which levels of visual information contributed to the clustered organization of categorical representations in the brain. To answer this question, the cortical representation of each category was dissected into multiple levels of representations, each of which was attributed to one single layer of features. For a given category, the features extracted from every exemplar of this category were kept only for one layer in the ResNet, while setting to zeros for all other layers. Through the above trained encoding models (see *Encoding models* in Materials and Methods), the single-layer visual features were projected onto a cortical map that only represented a certain level of visual information shared in the given category. The similarity and modularity in cortical representations of individual categories were then re-evaluated as a function of the layer in the ResNet. The layer with the highest modularity index contributed the most to the clustered organization in cortical categorical representation. The features encoded by this layer were visualized for more intuitive understanding of the types of visual information underlying the clustered organization. The feature visualization was based on an optimization-based technique^81^. Briefly, to visualize the feature encoded by a single unit in the ResNet, the input to the ResNet was optimized to iteratively maximize the output from this unit, starting from a Gaussian random pattern. Four optimized visualizations were obtained given different random initialization.

After obtained the layer-wise similarities in cortical representations of object categories, we further evaluated the correlation between the cortical similarity and the semantic similarity for each layer, and assessed its significance by using the aforementioned permutation test (p = 0.0001).

### Finer clustering of categorical representation

Considering object categories were defined hierarchically in semantics^79^, we asked how hierarchy of categorization^5^. More specifically, we tested whether the representational similarity and distinction in a larger spatial scale gave rise to a coarser level of categorization, whereas the representation in a smaller spatial scale gave rise to a finer level of categorization. To do so, we first examined the category representation in the scale of the entire visual cortex predictable by the encoding models, and clustered the categories into multiple clusters by using the clustering analysis of the representational similarity in this large scale. The resulting clusters of categories were compared with the superordinate-level semantic categories. Then, we focused on a finer spatial scale specific to the regions where category representations overlapped within each cluster. The cluster-specific region included the cortical locations whose activation was significantly higher for objects in the cluster compared to 50,000 random and non-selective objects (p < 0.01, two-sample t-test, Bonferroni correction). Given the spatial similarity of category representation in this finer scale, we defined sub-clusters within each cluster using the same clustering analysis as for the large-scale representation. The sub-clusters of categories were compared and interpreted against semantic categories in a finer level.

### Data availability

The datasets generated during and/or analyzed during the current study are available from the corresponding author on reasonable request.

## Electronic supplementary material

Supplementary Figures 1-5
