# Supplementary material for: Deep Residual Network Predicts Cortical Representation and Organization of Visual Features for Rapid Categorization
Source: Sci Rep. 2018 Feb 28;8:3752. doi: 10.1038/s41598-018-22160-9 (PMC5830584; doi:10.1038/s41598-018-22160-9)
Supplement: Supplementary file 1 — Supplementary Figures 1-5 [file 41598_2018_22160_MOESM1_ESM.docx]

**Supplementary Information** for

- **Deep Residual Network Predicts Cortical Representation and Organization of Visual Features for Rapid Categorization**
- Haiguang Wen, Junxing Shi, Wei Chen, Zhongming Liu
- **1. Supplementary Figure S1**


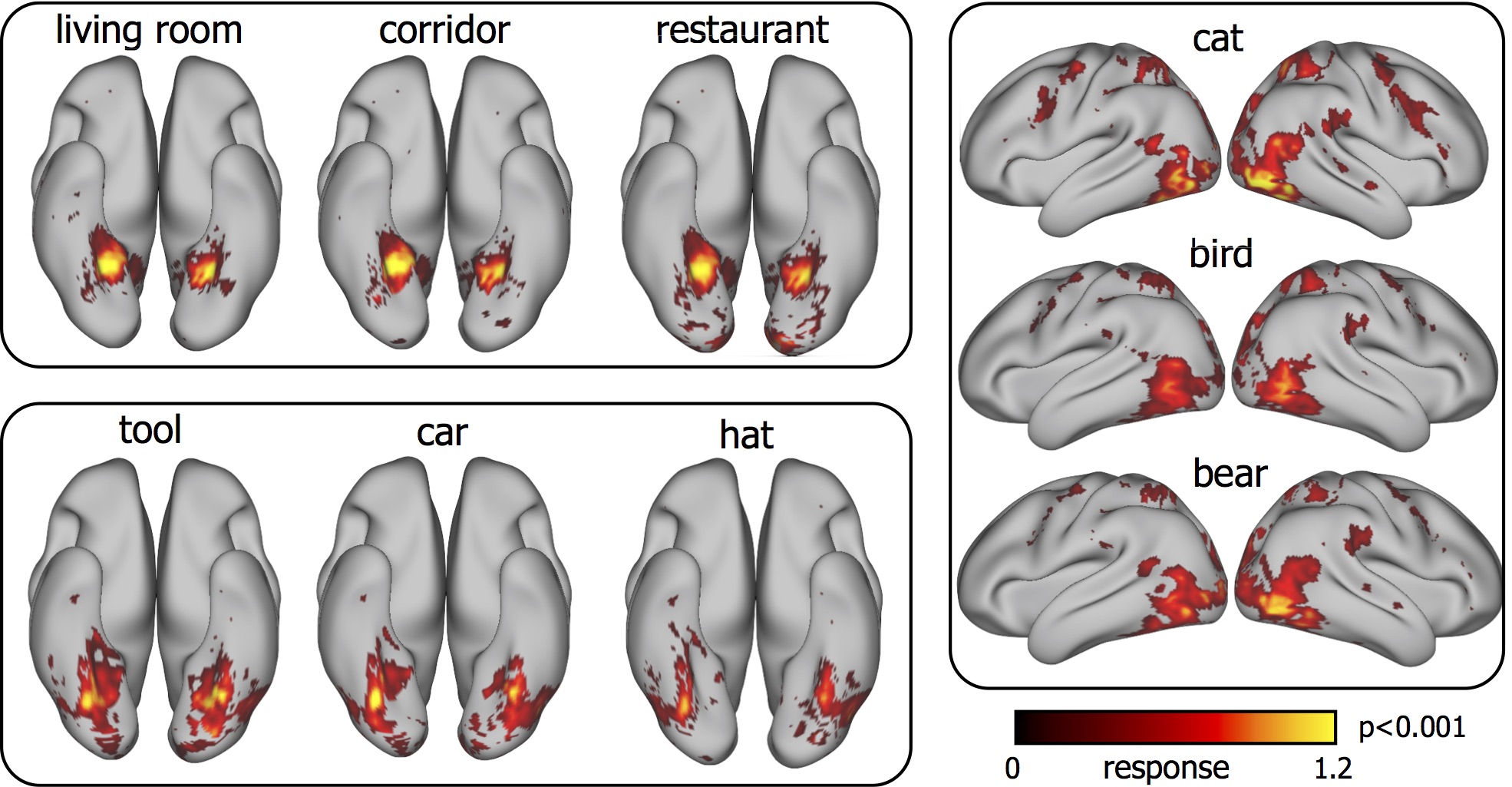


**Supplementary Figure S1. Example categories that shared similar cortical representations.** The cortical representations are displayed in best view on the inflated cortical surface. It was thresholded by assessing the significance of the response to a category against 50,000 random and non-selective natural pictures with two-sample t-test (p<0.001, Bonferroni correction for the number of voxels).

**2. Supplementary Figure S2**


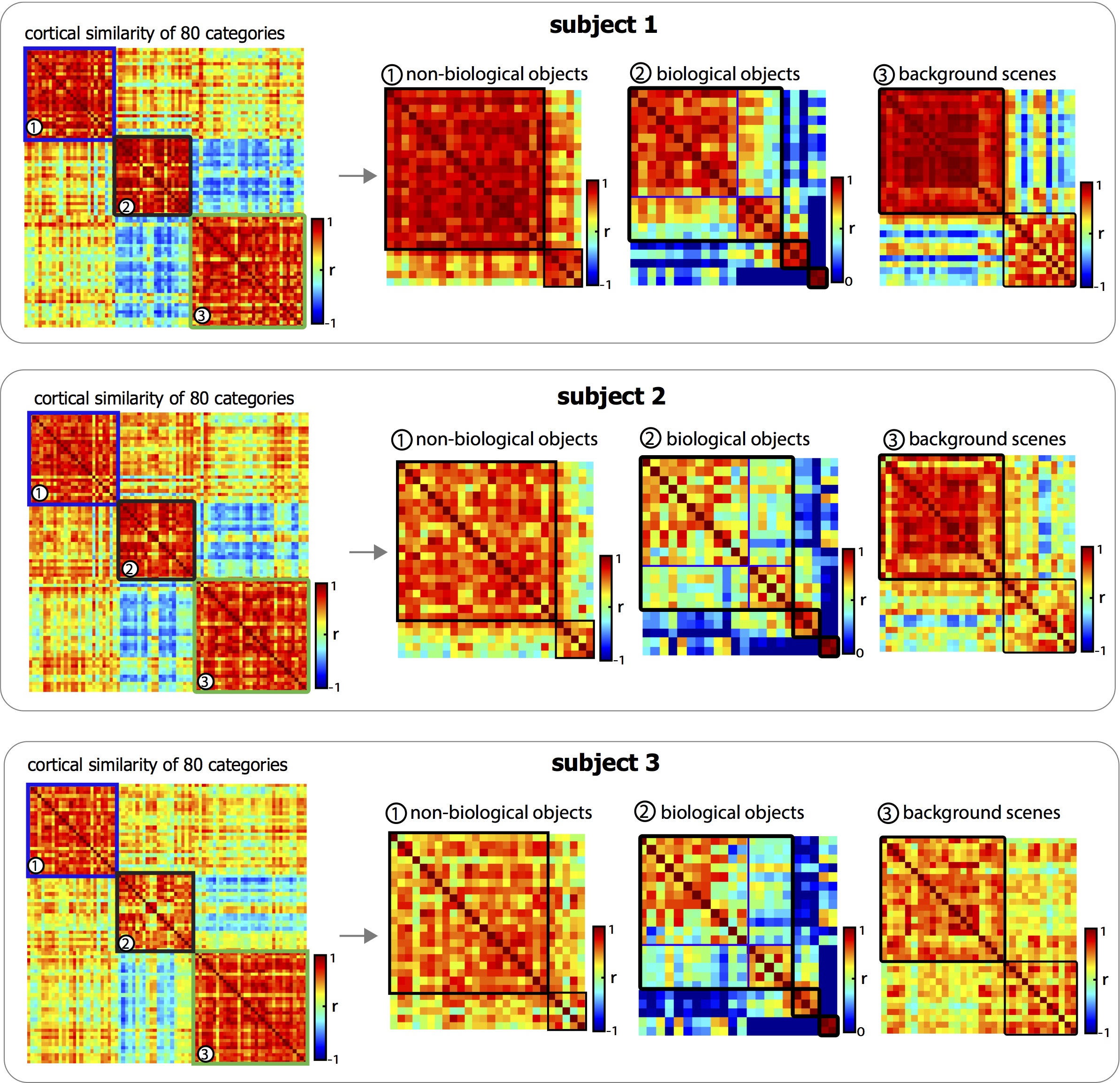


- **Supplementary Figure S2. Categorical hierarchy for individual subjects.** For each subject, the left shows the similarity between categories in the cortical representation at the scale of the entire visual cortex, and the order of categories is the same as in Figure 5.a. The right shows the similarity within each superordinate-level category in the finer-scale cortical representations, and the orders of categories are the same as in Figure 7.
- **3. Supplementary Figure S3**

- **Supplementary Figure S3. Correlation between the cortical similarity and the semantic similarity in sub-regions of the visual cortex.** For each region of interest (ROI), the bar shows the Pearson’s correlation between the inter-category similarity in the representational pattern and the inter-category similarity in the semantic meaning. The correlation is averaged across three different measures of the semantic similarity. * indicated p<0.0001 (permutation test).

**4. Supplementary Figure S4**


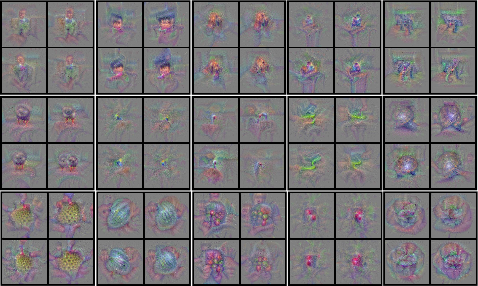


- **Supplementary Figure S4.** 15 example visual features at the 50^st^ layer in ResNet are visualized in pixel space. Each visual feature showed 4 exemplars that maximize the feature representation.

**5. Supplementary Figure S5**

- **Supplementary Figure S5. Semantic similarity matrix between 80 category labels.** The matrices show the similarity between the category labels. The order of the category and the cluster of the categories are the same as in Fig. 5a. The left shows the similarity measured by word2vec, and the right shows the similarity measured by GloVe. The similarity is calculated as the cosine distance of the vectors between every two categories.
